# Evaluation of an automated matching system of children and families to virtual mental health resources during COVID-19

**DOI:** 10.1186/s13034-024-00716-0

**Published:** 2024-02-09

**Authors:** Ronda F Lo, Anett Schumacher, Kaitlyn LaForge-Mackenzie, Katherine Tombeau Cost, Jennifer Crosbie, Alice Charach, Evdokia Anagnostou, Catherine S. Birken, Suneeta Monga, Daphne J. Korczak

**Affiliations:** 1 Department of Psychiatry, The Hospital for Sick Children, 555 University Ave, Toronto, ON M5G 1X8 Canada; 2 Department of Psychiatry, Temerty Faculty of Medicine, University of Toronto, Toronto, Canada; 3 Holland Bloorview Kids Rehabilitation Hospital, Toronto, ON Canada; 4 Department of Pediatrics, Faculty of Medicine, University of Toronto, Toronto, ON Canada; 5 Division of Paediatric Medicine, Hospital for Sick Children, Toronto, ON Canada

**Keywords:** Child, Adolescent, Mental health profiles, Resource matching

## Abstract

**Background:**

Children and their families often face obstacles in accessing mental health (MH) services. The purpose of this study was to develop and pilot test an electronic matching process to match children with virtual MH resources and increase access to treatment for children and their families during COVID-19.

**Methods:**

Within a large observational child cohort, a random sample of 292 families with children ages 6–12 years were invited to participate. Latent profile analysis indicated five MH profiles using parent-reported symptom scores from validated depression, anxiety, hyperactivity, and inattention measures: (1) Average Symptoms, (2) Low Symptoms, (3) High Symptoms, (4) Internalizing, and (5) Externalizing. Children were matched with virtual MH resources according to their profile; parents received surveys at Time 1 (matching process explanation), Time 2 (match delivery) and Time 3 (resource uptake). Data on demographics, parent MH history, and process interest were collected.

**Results:**

128/292 families (44%) completed surveys at Time 1, 80/128 families (63%) at Time 2, and a final 67/80 families (84%) at Time 3, yielding an overall uptake of 67/292 (23%). Families of European-descent and those with children assigned to the Low Symptoms profile were most likely to express interest in the process. No other factors were associated with continued interest or uptake of the electronic matching process. Most participating parents were satisfied with the process.

**Conclusions:**

The electronic matching process delivered virtual MH resources to families in a time-efficient manner. Further research examining the effectiveness of electronically matched resources in improving children’s MH symptoms is needed.

**Supplementary Information:**

The online version contains supplementary material available at 10.1186/s13034-024-00716-0.

## Introduction

Child and youth mental health (MH) declined during the COVID-19 pandemic [1–4]. The rising demand for child and youth MH services during the pandemic further exacerbated pre-existing difficulties with access to MH care, including cumbersome and non-streamlined referral processes, long wait lists, and the need for multiple points of assessment prior to receiving ongoing treatment [5–7].

In the face of rising demand for MH services with limited ability for in-person interactions, MH care rapidly shifted to virtual services [8, 9]. Although virtual MH services offer the potential for greater flexibility in service delivery, the problem of long wait times for assessments remains, owing to the shortage of child and adolescent psychiatrists and psychologists available to provide diagnostic assessments. In other medical disciplines, innovative processes to bypass the need for in-person assessments have begun to be implemented [10]. By employing machine learning techniques, for example, oncology providers in the United Kingdom further demonstrated that preparation time for radiotherapy could be reduced by 90%, dramatically accelerating the process to treatment initiation [11]. Thus, although many areas of MH care continue to rely on traditional assessments prior to treatment referral, some areas of healthcare have begun to expedite service delivery by incorporating data-driven approaches.

The purpose of this study was to pilot an innovative, data-driven process to enable rapid access to appropriate virtual MH resources for children based on parent-reported MH symptoms from online measures. Children were matched electronically to virtual MH resources based on their MH profile. We examined parents’ perceptions of the electronic match, including reasonability, acceptability, and satisfaction with the process as well as with the specific virtual MH resource with which their child was matched, to determine the initial feasibility of the process. We also examined rates of MH resource uptake with the matched resource, and whether child or clinical factors influenced feasibility or uptake.

## Method

### Participants

Data were collected as part of the Ontario COVID-19 and Kids Mental Health longitudinal study, a research collaborative of four established research cohorts with pre-existing participant bases: two clinically-referred pathways (i.e., MH, neurodevelopmental disorders diagnoses [NDD]) and two community-referred pathways. Clinical cohorts consisted of children who had been referred to an outpatient psychiatry clinic at a tertiary children’s hospital in Toronto, and/or participants of the Province of Ontario Neurodevelopmental Disorder network, for children with NDDs. The community cohort consisted of children and families recruited through an urban science museum. Detailed descriptions of the cohorts, consent, and participation processes of the larger project are provided elsewhere [12]. The present study included a subset of children ages 6 to 12 years to align with those of the matched resources or interventions. All participants provided informed written consent/assent to participate in the current study. The study was approved by the institutional research ethics boards at all participating institutions.

### Study process

This study occurred in two phases [1]: profile generation and [2] electronic matching.

#### Phase 1: profile generation

MH data were collected between February and August 2021, using the standardized measures below. When multiple assessments for an individual child were available, the most recent data were used to generate MH profiles. Using latent profile analysis (LPA) across the larger Ontario COVID-19 and Kids Mental Health longitudinal project (*n* = 1,608), five child MH profiles were indicated based on depression, anxiety, hyperactivity, inattention, and irritability scores (Table 1, Supplementary Table 1, Supplementary Fig. 1). Profiles were generated prior to the first study timepoint (Time 1).

**Table 1:** Child mental health profiles and matched mental health online resources

Profile	Profile Description	Mental Health Resource	Resource Description
**Average Symptoms**	Characterized by average scores on all mental health symptoms, with slightly above average scores on inattention and hyperactivity.	**Online Resources**	High-quality, age-appropriate online resources from reputable institutions regarding factors important for good Mental Health (e.g. sufficient and regular sleep, physical activity, positive interpersonal interactions) and practical strategies for optimizing these for families.
**Low Symptoms**	Characterized by average scores on depression and anxiety and below average scores on irritability, inattention, and hyperactivity.		
**Internalizing**	Characterized mostly by above average scores on depression, and slightly above average scores for, in descending order, anxiety, irritability, inattention, and hyperactivity. Although anxiety was not above average relative to the other symptoms, it was useful to cluster depression and anxiety together due to their high comorbidity rate and shared internalizing characteristics.	**Online Modified CBT Program**	Shortened online CBT program offered through the tertiary care children’s hospital in Toronto. Led by child psychologists trained in CBT.
**Externalizing**	Characterized by above average scores on inattention and hyperactivity, with above average depression, average anxiety, and slightly above average irritability scores.	**Parenting Intervention** **(ages 6–9 years)**	**Parenting Intervention**: Online “stepped” program at the tertiary care children’s hospital in Toronto, offering three stages of care for parents with children ages 6–9 and children with NDD diagnosis. Led by psychologists.
		Coping Power (ages 10–12 years)	
**High Symptoms**	Characterized by above average scores on all mental health symptom domains.	Parenting Intervention (ages 6–9 years)	**Coping Power**: Online individual and parent behavior management program offered at a large psychiatric hospital in Toronto for children and parents ages 10–12 years.
		**Coping Power** **(ages 10–12 years)**	

#### Phase 2: electronic match process

**Fig. 1:**
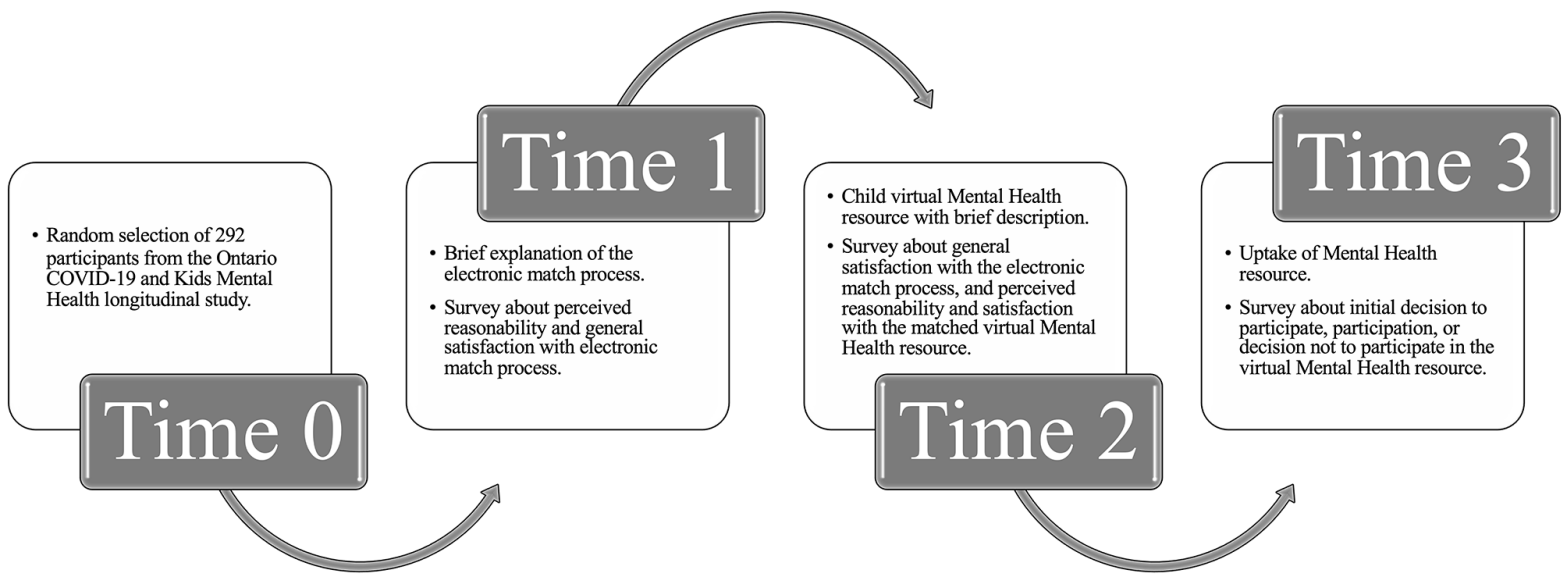
Overview of the electronic match process

Three hundred families from the larger Ontario COVID-19 and Kids Mental Health longitudinal project were randomly selected to participate in the present study, and 292 families consented. Online surveys were distributed to parents at three time points (Fig. 1), using REDCAP electronic data capture tools [13, 14].

At Time 1, parents were provided with a brief explanation of how their child’s profile was created and that a virtual MH resource would be offered based on this profile. Parents completed the Time 1 survey, regarding their perception of the feasibility of an electronic matching process, prior to receiving the virtual MH resource.

Following completion of the Time 1 survey, parents received their child’s matched virtual MH resource with a brief description of the resource. The resources provided for each MH profile are described in Table 1. Following receipt of the matched resource and description (Time 2), parents completed a second survey about their perceptions of the electronic match and the matched virtual MH resource (i.e., satisfaction with the electronic match process, perceived reasonability and satisfaction with the matched virtual MH resource). Following the Time 2 survey, parents were provided with access to either the resource package (Average and Low Symptoms profiles), or with the contact information for enrollment in the matched resource (Internalizing, Externalizing, and High Symptom profiles).

One month after completion of Time 2, a further study follow-up (Time 3) was conducted to measure uptake of the matched resource.

### Measures

#### Demographic measures

Child age, sex at birth, ethnicity, pre-existing MH/NDD diagnosis, and family annual household income were collected.

#### Child MH measures

Depression was measured with the Major Depressive Disorder subscale of the Revised Child Anxiety and Depression Scales-Parent Version (RCADS-P) and t-scores were computed [15]. Anxiety was measured with the 9-item Generalized Anxiety Disorder subscale (GAD-9) of the Screen for Child Anxiety Related Disorders (SCARED) instrument [16, 17]. Hyperactivity and inattention were measured with the respective subscales of the Strengths and Weaknesses of Attention-Deficit/Hyperactivity Disorder Symptoms and Normal Behavior Scale (SWAN) [18]. Irritability was measured with the 6-item subscale from The Irritability and Dysregulation of Emotions Questionnaire (TIDES) [19].

#### Parent MH measures

Parents’ self-reported depression and anxiety symptoms were assessed with the 8-item version of the Patient Health Questionnaire (PHQ-8) and the Generalized Anxiety Disorder-7 (GAD-7) measure, respectively [20, 21].

#### Feasibility measures

The four-item Acceptability of Intervention Measure (AIM) and the Intervention Appropriateness Measure (IAM) measures [22] were combined following principal components analysis to assess the perceived reasonability of the electronic match process and virtual MH resource. Mean composite scores were computed for all measures. Perceived satisfaction with the electronic match process was assessed using the 7-item Program Feedback Scale and the 8-item Client Satisfaction Questionnaire for Internet-based Interventions (CSQ-I) [23, 24]. The Readiness for Therapy Questionnaire (RTQ) was adapted to assess how ready parents were to undertake actions for their child’s intervention [25]. Further details about the reasonability, satisfaction, and readiness for therapy, including psychometrics in the current sample, are included in the Supplementary Methods.

#### Uptake of virtual MH Resource

Parents were asked to report the extent to which they participated or intended to participate in the virtual MH resource matched to their child [1]: enrolled (completed resource, or signed up and completion of resource in progress) [2], waitlisted for resource [3] considering enrollment but not signed up and [4] will not enroll (no intention).

### Data analysis

#### Phase 1: child MH profiles

LPA was used to identify child MH profiles across the larger Ontario COVID-19 and Kids Mental Health longitudinal project (*n* = 1,608). LPA is a data-driven technique that identifies latent subgroups within a sample, based on similar responses to a set of continuous variables. The LPA was conducted with MPlus using full information maximum likelihood estimator. Variances were constrained to be equal across profiles, and covariances between profiles were constrained to zero. LPAs computed posterior classification probabilities that indicated a child’s probability of being assigned to each profile with the aim of achieving a minimum probability of 0.80 [26]. Further details regarding profile assignment are provided in the Supplementary Methods.

#### Phase 2: completion at each time point

Potential factors that predict Time 1 completion were tested using logistic regression. These factors included child’s assigned profile as well as demographic variables: age, sex at birth, ethnicity, household income, and pre-existing MH/NDD diagnosis. There were four dummy-coded variables for profile, with the Average Symptoms profile serving as the reference group for all four dummy variables. Subsequent logistic regression examined whether demographics, perceived reasonability, or satisfaction at Time 1 predicted completion at Time 2. Further logistic regression examined whether demographics, perceived reasonability and satisfaction at Time 2, readiness for therapy at Time 2, or the matched virtual MH resource at Time 2 predicted uptake of the virtual MH resources at Time 3.

## Results

A total of 292 families with children ages 6–12 years were recruited for this study from age-appropriate participants of the Ontario COVID-19 and Kids Mental Health longitudinal project (*n* = 1,608). Children were a mean age of 8.2 ± 1.7 years, and 46% were female. The majority of children did not have a pre-existing MH/NDD diagnosis (59%). The proportion of children in each profile was as follows: Average Symptoms (*n* = 101, 34.6%), Low Symptoms (*n* = 38, 13%), Internalizing (*n* = 73, 25%), Externalizing (*n* = 55, 18.8%), High Symptoms (*n* = 25, 8.6%). Further details regarding participant characteristics are described in Table 2.

**Table 2:** Participant characteristics by child mental health profile

Total: *n* = 292	Average Symptoms	Low Symptoms	Internalizing	Externalizing	High Symptoms
Sample (*n*)	101	38	73	55	25
Age – *M* (*SD*)	7.97 (1.76)	8.45 (1.98)	8.11 (1.76)	8.65 (1.57)	8.20 (1.35)
Sex at birth - *%* (*n*)					
Male	51.5% (52)	44.7% (17)	52.1% (38)	63.6% (35)	60.0% (15)
Female	48.5% (49)	55.3% (21)	48.0% (35)	36.4% (20)	36.0% (9)
Did not respond	-	-	-	-	4.0% (1)
Income - *%* (*n*)					
Low (< 80k)	18.8% (19)	15.8% (6)	19.2% (14)	38.2% (21)	36.0% (9)
High ( > = 80k)	56.4% (57)	57.9% (22)	61.6% (45)	47.3% (26)	48.0% (12)
Did not respond	24.8% (25)	26.3% (10)	19.2% (14)	14.5% (8)	16.0% (4)
Ethnicity - *%* (*n*)					
European	60.4% (61)	44.7% (17)	68.5% (50)	54.5% (30)	80.0% (20)
Non-European	11.9% (12)	23.7% (9)	19.2% (14)	27.3% (15)	4.0% (1)
Mixed	25.7% (26)	29.0% (11)	12.3% (9)	16.4% (9)	12.0% (3)
Did not respond	2.0% (2)	2.6% (1)	-	1.8% (1)	4.0% (1)
Diagnosis of Mental Health Disorder - *%* (*n*)					
No	81.2% (82)	86.8% (33)	47.9% (35)	29.1% (16)	24.0% (6)
Yes	18.8% (19)	13.2% (5)	52.1% (38)	70.9% (39)	76.0% (19)

### Predicting rates of completion at each time point in phase 2

#### Time 1 completion

Parents of 128 out of 292 (44%) children were interested in obtaining virtual MH resources for their child (completed Time 1). Ethnicity, MH profile, and most recently completed survey wave predicted interest in the electronic process (Table 3). Parents with children assigned to the Low Symptoms profile were 3 times more likely to express initial interest in the matching process compared with children assigned to the Average Symptoms profile (OR = 3.12, 95% CI [1.19, 8.55], *p* = .023). Parents from European descent families were 3.5 times more likely to express initial interest in the electronic match process compared with children from non-European descent families (OR = 0.28, 95% CI [0.12, 0.61], *p* = .002). Finally, recent engagement in the larger longitudinal study also predicted initial interest in the electronic process, such that parents who completed the most recent survey were 3 times more likely to express interest in participating in the electronic match process compared with those who had completed the least recent survey (OR = 3.43, 95% CI [1.24, 13.46], *p* = .033).

**Table 3:** Predictors for completion and uptake at each time point

	Time 1 Completion (*n* = 128)			Time 2 Completion (*n* = 80)			Time 3 Uptake (*n* = 67)		
Variable	OR	95% CI	*p*	OR	95%	*p*	OR	95%	*p*
Age	1.09	0.92, 1.30	0.309	0.87	0.66, 1.14	0.315	2.97	1.22, 12.21	.**049**
Sex at birth (Male = 0)									
Female	1.07	0.60, 1.91	0.825	2.33	0.94, 6.09	0.075	1.69	0.13, 25.59	0.686
Income (Low = 0)									
High	0.76	0.39, 1.46	0.413	1.15	0.41, 3.17	0.792	0.30	0.01, 6.27	0.470
Ethnicity (European = 0)									
Non-European	0.28	0.12, 0.62	**0.002**	1.72	0.42, 8.98	0.476	0.79	0.09, 9.21	0.834
Mixed	0.53	0.24, 1.17	0.122	1.11	0.33, 3.91	0.872	-	-	-
Diagnosis of Mental Health Disorder (No = 0)									
Yes	1.28	0.65, 2.52	0.472	1.87	0.66, 5.57	0.245	0.24	0.02, 2.36	0.249
Profile (Average Symptoms = 0)									
Low Symptoms	3.12	1.19, 8.55	**0.023**	0.44	0.11, 1.70	0.238	-	-	-
Internalizing	1.21	0.55, 2.68	0.634	0.59	0.16, 2.08	0.414	-	-	-
Externalizing	1.27	0.52, 3.11	0.596	0.68	0.16, 2.73	0.585	-	-	-
High Symptoms	1.62	0.51, 5.30	0.415	0.28	0.05, 1.37	0.121	-	-	-
Last completed survey	3.43	1.24, 13.46	**0.033**	-	-	-	-	-	-
Perceived reasonability of EMP at T1				0.66	0.20, 1.99	0.466	-	-	-
Perceived satisfaction of EMP at T1				1.62	0.50, 5.54	0.425	-	-	-
VMR (Online Resources = 0)									
Online Modified CBT							0.16	0.00, 3.14	0.234
Parenting Intervention							0.03	0.00, 1.98	0.150
Coping Power							0.03	0.00, 1.14	0.104
Perceived reasonability of VMR at T2							3.91	0.30, 76.54	0.292
Perceived satisfaction of VMR at T2							0.15	0.00, 2.53	0.221
Readiness for therapy at T2							5.75	1.13, 72.67	0.079

Note. CBT = Cognitive Behavioural Therapy; EMP = Electronic Match Process; VMR = Virtual Mental Health Resource

#### Time 2 completion

Parents that completed Time 1 measures (*n* = 128) perceived the electronic match process to be somewhat reasonable (*M* = 3.79, *SD* = 0.65, range = 0–5) and felt somewhat satisfied with the proposed process (*M* = 2.81, *SD* = 0.54, range = 0–5). Of these 128 parents, 80 completed Time 2 assessments to receive their child’s MH profile and electronic MH resource match assignment (63% uptake). Neither demographic variables, MH profile, nor perceived reasonability and satisfaction with the electronic match process at Time 1 predicted participation at Time 2 (Table 3).

#### Time 3 completion (uptake of resources)

Parents who received the information regarding their child’s MH profile and suggested MH resource match at Time 2 (*n* = 80) perceived their child’s matched virtual MH resource to be somewhat reasonable (*M* = 3.70, *SD* = 0.63, range = 0–5), and were somewhat satisfied with the resource (*M* = 2.80, *SD* = 0.54, range = 0–5), consistent with their previous reports about the electronic match process. Overall, parents felt somewhat ready for treatment for their child at Time 2 (*M* = 2.43, *SD* = 0.78, range = 0–5). Out of the 80 families who received their child’s MH profile and electronic MH match assignment, 67 (84%) had either engaged in the MH resource or were on the waiting list, resulting in an overall uptake rate of 67/292 (23%) (Table 3). The only significant predictor of uptake of matched MH resource was child age, with families of older children being more likely to engage in the matched MH resource (OR = 2.97, 95% CI [1.22, 12.21], *p* = .049; see Table 3).

#### Predicting parents’ perceptions of the MH resource and parent’s readiness for therapy

Perception of the match process at Time 1 and satisfaction with the matched MH resource at Time 2 were not predicted by any of the included factors measured (Supplementary Table 2). Matching to the individual and parent behaviour management program (i.e., the matched resource for older children in the Externalizing and High Symptoms profiles) was associated with decreased family satisfaction with the matching process (*b* = -0.61, 95% CI [ -1.13, -0.09], *p* = .023).

In examining potential predictors of readiness for therapy, only parent’s self-reported depression was found to be significant, with higher parent depression scores associated with greater readiness for therapy for their child (OR = 1.2, 95% CI [ 0.02, 0.35], *p* = .033; Table 2).

## Discussion

This study examined whether matching children to virtual MH resources electronically based on online, parent-reported MH symptoms was an acceptable and feasible strategy for accessing scarce children’s MH resources. The results indicate that the electronic matching process to MH resources may be an accessible and more timely alternative to traditional referral to MH services. Of interested families, 23% ultimately engaged in uptake of the matched virtual MH resource.

Once families enrolled in the study, retention rates increased throughout the study. More specifically, of the 44% of invited families that were interested in having their child matched to a virtual MH resource, 63% received the MH profile and the electronically matched virtual MH resource, and 84% of families that received the matched resource participated in the assigned MH resource. Thus, factors that promote initial engagement with the process are important to consider. We found that parents of European descent, relative to non-European descent, were more likely to express initial interest in the virtual MH resource for their child. Ethnicity and culture may play a key role in initial engagement with MH interventions, with ethnically minoritized groups less likely to express interest [27]. Thus, future research examining ways to facilitate diversity-inclusive processes to increase access to MH resources through data-driven approaches is needed. Furthermore, families of children who experienced low levels of MH symptoms (i.e., children who were assigned to the Low Symptoms profile) and families who were more recent research participants were more likely to show interest in the virtual MH resource, indicating the importance of parent engagement in this process. Parents play an important role in their child’s MH, with research showing that parental awareness of MH concerns increases identification of MH problems in children [28]. Thus, increasing parental involvement may lead to elevated interest in participation in an electronic matching process.

Uptake of the matched MH resource was modest, with 23% of interested families engaging in the matched intervention. Prior comparable research with which to compare these findings is scant. In a study by Reid and colleagues, families were able to self-refer to virtual MH assessments online, which were conducted face to face by pediatric emergency department physicians [29]. Acceptability of this process was found to be low by both caregivers and physicians, owing to insufficient time and number of MH clinicians to provide adequate assessments [30]. In contrast, children and their families in our study did not require in-person assessment. While the fully electronic/online process removed this potential bottleneck to access to care, the lack of human contact may have been a deterrent to engaging in the matched MH resource. Thus, future research using qualitative approaches is needed to gain better understanding of the reasons for children and their families to participate in the matched MH resource.

Results showed that parents whose children were assigned to the individual and parent behaviour management program perceived the matched MH resource to be less reasonable relative to parents whose children were matched to other MH resources. This suggests that satisfaction with the matching process may be different depending on either the specific program to which families were matched, or the specific MH problem for which treatment is offered. Resource-specific factors that may have influenced the acceptability and uptake of the program in the current study included administration through a MH hospital for children and adults, rather than through a general children’s hospital, as was the case for the other interventions. As MH stigma is one of the biggest factors that deter children and families from accessing treatment [31], the stigma associated with a non-children’s MH hospital might have led to a negative perception of this resource. On the other hand, factors associated with the specific MH profile matched to this resource may also have contributed to the decreased acceptance and uptake of the program, as this group included older children categorized as Externalizing (increased disruptive behaviour) and High Symptoms (elevated symptoms across multiple domains) that were matched to this resource. This suggests that in certain situations in-person contact with a clinician or specialist may be helpful to provide important context and information for families. Families with children exhibiting increased externalizing symptoms alone or in combination with increased internalizing symptoms may require some degree of person to person communication at the assessment or referral stages to enhance understanding of their child’s MH profile, the suggested treatment resource, and to provide greater support connecting with MH care.

Lastly, our study showed that parents with greater depression symptoms were more eager for their child to receive treatment, indicating the potential role of parental distress in accessing MH treatment for their child. As children of depressed parents are at increased risk for developing depression [32], it may be that depressed parents have increased sensitivity regarding their child’s MH symptoms, and greater interest in early detection of possible MH problems in order to facilitate early intervention. However, this requires further investigation in future studies.

### Limitations

Despite the strengths of this study examining a novel data-driven approach to child MH service access, there are also limitations to consider. Although the sample size of the current study was reasonably large for a pilot study, it represents only a single iteration of the matching process. Thus, further development of the electronic matching approach is required to examine multiple additional perspectives (for example, child and clinician views) to maximize feasibility and examine potential cost and time-efficiency of the proposed approach. Also, as this study was focused on feasibility, efficacy of the electronically matched MH resource was not evaluated. Future research examining the effectiveness of electronically matched MH services using the proposed approach is needed to ensure that the matched services ultimately improve child MH outcomes.

## Conclusion

In summary, this pilot study describes a novel, data-driven approach to increasing access to MH care for children during the COVID-19 pandemic, a time of increased MH service need and rapid transition to virtual MH care. We found that an electronic matching process to virtual child MH resources, based on parent-report of child MH symptoms, was feasible and acceptable to parents of 6- to 12-year-old children experiencing a wide range of MH symptoms. Further research is needed to maximize feasibility across all patient populations, symptom profiles and services, elicit feedback from stakeholders more broadly, and determine whether interventions undertaken based on an electronic matching process are effective in improving children’s MH outcomes.

### Electronic supplementary material

Below is the link to the electronic supplementary material.

Supplementary Material 1

## Data Availability

The data that support the findings of this study are not openly available and are available from the corresponding author upon reasonable request (including a study outline), subject to review.

## Abbreviations

AIM: Acceptability of Intervention Measure
CBT: Cognitive Behavioural Therapy
CSQ-I: Client Satisfaction Questionnaire for Internet-based Interventions
GAD-7: Generalized Anxiety Disorder – 7
GAD-9: Generalized Anxiety Disorder – 9
IAM: Intervention Appropriateness Measure
LPA: Latent Profile Analysis
MH: Mental Health
NDD: Neurodevelopmental Disorder Diagnoses
PHQ-8: Patient Health Questionnaire – 8
RCADS-P: Revised Child Anxiety and Depression Scales – Parent Version
RTQ: Readiness for Therapy Questionnaire
SCARED: Screen for Child Anxiety Related Disorders
SWAN: Strengths and Weaknesses of Attention-Deficit/Hyperactivity Disorder Symptoms and Normal Behavior Scale
TIDES: The Irritability and Dysregulation of Emotions Questionnaire
