# Supplementary material for: Evaluation of an automated matching system of children and families to virtual mental health resources during COVID-19
Source: Child Adolesc Psychiatry Ment Health. 2024 Feb 9;18:24. doi: 10.1186/s13034-024-00716-0 (PMC10858567; doi:10.1186/s13034-024-00716-0)
Supplement: Supplementary file 1 — Supplementary Material 1 [file 13034_2024_716_MOESM1_ESM.docx]

**Supplementary Material**

**Supplementary Methods**

**Feasibility Measures**

***Reasonability of Electronic Match Process and Virtual MH Resource***

The Acceptability of Intervention Measure (AIM) and the Intervention Appropriateness Measure (IAM) were combined to assess the perceived reasonability of the electronic match process and virtual MH resource (1). The word “intervention” in the scales was replaced with “electronic match process” or with the name of the assigned virtual MH resource, to measure perceived reasonability of the electronic match process or virtual MH resource, respectively. Each measure is comprised of four items, each scored on a 5-point Likert scale ranging from 1 (completely disagree) to 5 (completely agree). Principal components analyses (PCA) revealed that combining the AIM and IAM resulted in one component emerging, which was labeled as “perceived reasonability”. Reliabilities for perceived reasonability for the electronic match process (α = .94) and virtual MH resource (α = .96) were excellent. A mean composite was created from both the AIM and IAM for analyses.

***Satisfaction of Electronic Match Process and Virtual MH Resource***

Participants were asked about their perceived satisfaction with the electronic match process with the Program Feedback Scale (2), a 7-item measure scored using a 5-point Likert scale, from 1 (really disagree) to 5 (totally agree), which showed good reliability (α = .88). The Client Satisfaction Questionnaire for Internet-based Interventions (CSQ-I) was adapted for the purpose of assessing the participants’ satisfaction with their virtual MH resource (3). The 8-item scale is scored on a 4-point Likert-type scale with response options ranging from 1 (poor) to 4 (excellent), which showed excellent reliability (α = .91). Mean composite scores were computed for all measures.

***Readiness for Therapy***

The Readiness for Therapy Questionnaire (RTQ) was originally used to assess how ready parents were to undertake actions for their child’s intervention (4). A PCA was conducted given that the original article did not report validity or reliability measures, reported poor sensitivity and specificity, and assumed the scale was one-factor. The PCA revealed two components (“ready” and “rigid” or not ready for change) with mediocre Cronbach alphas for each factor (“ready”: α = .56; “rigid”: α = .58). As the measure was found to perform inadequately, an alternate measurement strategy was applied. Four currently practicing child psychiatrists were polled to choose the item they felt best represented readiness for change. Two items emerged, with ultimately full agreement on one item representing perseverance (i.e., “Even if the resource becomes difficult, I will stick with the resource until the end.”). As such, this item was used to measure parent and child readiness for therapy.

**Data Analysis**

***Phase 1: Child MH profiles***

LPA was used to identify child MH profiles across the larger Ontario COVID-19 and Kids Mental Health longitudinal project (*n* = 1,608). Selecting the correct LPA model required examining several fit indices while also considering parsimony and interpretability. Five profiles were selected as the best fitting model, based on several fit indices (Supplementary Table 1), interpretability of profiles, and feasibility of matching virtual MH resources for profiles. Profiles revealed by LPA were latent, not observed. LPAs computed posterior classification probabilities that indicated a child’s probability of being assigned to each profile. The model assigned each child with a profile even if their posterior classification probabilities did not clearly reflect a profile. For example, if a child had a posterior classification probability of 0.50 for Average Symptoms, 0.35 for Externalizing, and 0.15 for Low Symptoms, the model automatically assigned the Average Symptoms profile because it represented the largest probability. It was therefore possible that an individual child was assigned a profile that was not particularly reflective of their MH symptoms. Initial evidence of this was observed by the relatively low entropy score (0.71; ideally, this should be above 0.80) (5). As children assigned to the Average or Low Symptoms MH profiles were matched to online resources, a child who was misclassified as Average or Low MH profile, and might otherwise be more suited to another profile, may not receive the resource best suited to their needs. Therefore, we examined all posterior classification probabilities of children assigned to the Average and Low Symptoms MH profiles. If the child did not have at least a 0.80 probability for any profile and/or lacked an obvious profile, their raw MH scores were manually checked. Children with clinical-levels or subclinical-levels of MH scores were re-assigned to the closest fitting profile. As a result of this process, a total of 68 children were re-assigned from Low (*n* = 6) and Average Symptoms (*n* = 62) profiles to the Internalizing, Externalizing, and High Symptoms MH profiles.

**Supplementary Table 1.** LPA Fit Indices

| Number of Profiles | AIC | BIC | Entropy | Comparing Models | BLRT | aLMR (LRT) |
| --- | --- | --- | --- | --- | --- | --- |
| 2 | 53130.16 | 53217.14 | 0.705 | *2 vs 1* | -27137.431*** | 1150.903*** |
| 3 | 52672.61 | 52792.21 | 0.703 | *3 vs 2* | -26549.081*** | 459.257** |
| 4 | 52248.50 | 52400.70 | 0.714 | *4 vs 3* | -26314.306*** | 426.557*** |
| **5** | **52054.03** | **52238.85** | **0.710** | ***5 vs 4*** | **-26096.247***** | **201.943*** |
| 6 | 51932.95 | 52150.394 | 0.735 | *6 vs 5* | -25993.013*** | 130.155 |

*Note*. Bolded row indicates the final number of profiles selected. AIC and BIC are relative fit indices, and smaller numbers indicate better fit vs. model parsimony trade-off. Entropy is a weighted average of posterior classification probabilities and values closer to 1 indicate better fit (0.80 is a commonly used cut-off). BLRT and aLMR are bootstrapped and adjusted Lo-Mendell-Rubin likelihood ratio tests, respectively, and both compare nested models (k+1 profile vs. k profile). Statistical significance suggests the k+1 profile has better fit than the k profile model. *** = < .001, ** = < .01, * < .05**Supplementary Table 2.** Predictors for Parents’ Perceptions and Readiness for Therapy

|  | **Reasonability of EMP at Time 1** | | **Satisfaction of**  **EMP at Time 1** | | **Reasonability of VMR at Time 2** | | **Satisfaction of**  **VMR at Time 2** | | **Readiness for Therapy at Time 2** | | |
| --- | --- | --- | --- | --- | --- | --- | --- | --- | --- | --- | --- |
| **Variable** | *b* (*SE*) | *p* | *b* (*SE*) | *p* | *b* (*SE*) | *p* | *b* (*SE*) | *p* | *OR* | *95% CI* | *p* |
| Age | -0.003 (0.04) | .947 | -0.05 (0.04) | .224 | -0.05 (0.05) | .307 | -0.02 (0.04) | .674 | 0.75 | -0.65, 0.06 | .109 |
| Sex at birth (Male = 0) |  |  |  |  |  |  |  |  |  |  |  |
| Female | 0.21 (0.13) | .107 | 0.01 (0.13) | .907 | -0.02 (0.18) | .923 | 0.08 (0.16) | .634 | 1.12 | -1.10, 1.33 | .852 |
| Income (Low = 0) |  |  |  |  |  |  |  |  |  |  |  |
| High | 0.03 (0.15) | .853 | -0.02 (0.14) | .876 | 0.04 (0.20) | .850 | 0.25 (0.18) | .170 | 0.58 | -1.95, 0.85 | .444 |
| Ethnicity (European = 0) |  |  |  |  |  |  |  |  |  |  |  |
| Non-European | -0.18 (0.20) | .360 | 0.04 (0.19) | .852 | 0.14 (0.24) | .580 | 0.14 (0.21) | .513 | 0.23 | -3.17, 0.15 | .078 |
| Mixed | -0.04 (0.18) | .826 | 0.02 (0.17) | .901 | -0.15 (0.23) | .533 | 0.06 (0.20) | .778 | 0.62 | -2.12, 1.11 | .557 |
| Diagnosis of Mental Health Disorder (No = 0) |  |  |  |  |  |  |  |  |  |  |  |
| Yes | 0.07 (0.15) | .639 | 0.09 (0.15) | .553 | 0.21 (0.19) | .258 | 0.07 (0.16) | .681 | 2.67 | -0.27, 2.28 | .128 |
| Profile  (Average Symptoms = 0) |  |  |  |  |  |  |  |  |  |  |  |
| Low Symptoms | -0.005 (0.20) | .981 | 0.03 (0.19) | .893 | - | - | - | - | - | - | - |
| Internalizing | -0.28 (0.18) | .114 | -0.06 (0.17) | .744 | - | - | - | - | - | - | - |
| Externalizing | -0.06 (0.20) | .774 | -0.03 (0.20) | .898 | - | - | - | - | - | - | - |
| High Symptoms | -0.06 (0.24) | .809 | -0.19 (0.23) | .419 | - | - | - | - | - | - | - |
| VMR  (Online Resources = 0) |  |  |  |  |  |  |  |  |  |  |  |
| Online Modified CBT |  |  |  |  | 0.03 (0.23) | .893 | -0.02 (0.21) | .905 | 1.77 | -1.05, 2.20 | .487 |
| Parenting Intervention |  |  |  |  | -0.04 (0.32) | .899 | -0.0002 (0.28) | .999 | 2.66 | -1.03, 3.08 | .345 |
| Coping Power |  |  |  |  | -0.61 (0.26) | **.023** | -0.36 (0.23) | .116 | 0.38 | -2.77, 0.76 | .273 |
| Parent Depression |  |  |  |  | -0.01 (0.02) | .532 | 0.02 (0.02) | .373 | 1.20 | 0.02, 0.35 | **.033** |
| Parent Anxiety |  |  |  |  | 0.03 (0.03) | .224 | 0.01 (0.02) | .824 | 0.83 | -0.37, 0.00 | .056 |

*Note.* CBT = Cognitive Behavioural Therapy; EMP = Electronic Match Process; VMR = Virtual Mental Health Resource. Outcomes measured at Time 1 and Time 2 had n = 128 and n = 80, respectively.

**Supplementary Figure 1:** Latent profile analysis across the entire Ontario COVID-19 and Kids Mental Health longitudinal study.


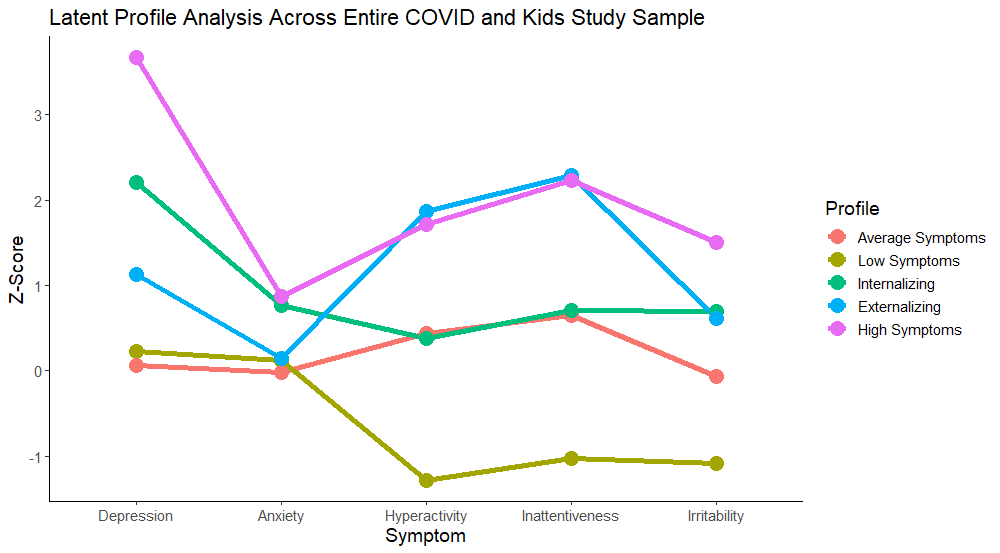


**References**

1. Weiner BJ, Lewis CC, Stanick C, Powell BJ, Dorsey CN, Clary AS, et al. Psychometric assessment of three newly developed implementation outcome measures. Implementation Science [Internet]. 2017 Dec 29;12(1):108. Available from: https://implementationscience.biomedcentral.com/articles/10.1186/s13012-017-0635-3

2. Schleider JL, Mullarkey MC, Weisz JR. Virtual Reality and Web-Based Growth Mindset Interventions for Adolescent Depression: Protocol for a Three-Arm Randomized Trial. JMIR Res Protoc [Internet]. 2019 Jul 9;8(7):e13368. Available from: https://www.researchprotocols.org/2019/7/e13368/

3. Boß L, Lehr D, Reis D, Vis C, Riper H, Berking M, et al. Reliability and Validity of Assessing User Satisfaction With Web-Based Health Interventions. J Med Internet Res [Internet]. 2016 Aug 31;18(8):e234. Available from: http://www.jmir.org/2016/8/e234/

4. Ghomi M, Wrightman M, Ghaemian A, Grey N, Pickup T, Richardson T. Development and validation of the Readiness for Therapy Questionnaire (RTQ). Behavioural and Cognitive Psychotherapy [Internet]. 2021 Jul 17;49(4):413–25. Available from: https://www.cambridge.org/core/product/identifier/S1352465820000764/type/journal_article

5. Tein JY, Coxe S, Cham H. Statistical Power to Detect the Correct Number of Classes in Latent Profile Analysis. Struct Equ Modeling [Internet]. 2013 Oct;20(4):640–57. Available from: http://www.tandfonline.com/doi/abs/10.1080/10705511.2013.824781
